# Crystal structure of 2-ethyl-3-(4-fluoro­phenyl­sulfin­yl)-5,7-dimethyl-1-benzo­furan

**DOI:** 10.1107/S1600536814019023

**Published:** 2014-08-30

**Authors:** Hong Dae Choi, Uk Lee

**Affiliations:** a Department of Chemistry, Dongeui University, San 24 Kaya-dong, Busanjin-gu, Busan 614-714, Republic of Korea; b Department of Chemistry, Pukyong National University, 599-1 Daeyeon 3-dong, Nam-gu, Busan 608-737, Republic of Korea

**Keywords:** crystal structure, benzo­furan, 4-fluoro­phen­yl, π–π inter­actions, sulfinyl group, natural products

## Abstract

In the title compound, C_18_H_17_FO_2_S, the dihedral angle between the planes of the benzo­furan ring system (r.m.s. deviation = 0.004 Å) and the 4-fluoro­phenyl ring is 86.38 (6)°. The terminal C atom of the ethyl substituent is displaced by 1.444 (3) Å from the benzo­furan ring system to the same side of the mol­ecule as the 4-fluoro­phenyl ring. In the crystal, mol­ecules are linked *via* pairs of C—H⋯π inter­actions into inversion-related dimers. These dimers are further linked by π–π inter­actions between the benzene rings of neighbouring mol­ecules [centroid–centroid distance = 3.715 (3) Å] and between the furan rings of neighbouring mol­ecules [centroid–centroid distance = 3.598 (3) Å]. The mol­ecules are stacked along the *a*-axis direction. In the sulfinyl group, the S and O atoms are disordered over two sets of sites, with site-occupancy factors of 0.797 (3) and 0.213 (3).

## Related literature   

For pharmaceutical properties of benzo­furan compounds, see: Aslam *et al.* (2009 ▶); Galal *et al.* (2009 ▶); Howlett *et al.* (1999 ▶); Khan *et al.* (2005 ▶); Ono *et al.* (2002 ▶). For natural products with a benzo­furan ring, see: Akgul & Anil (2003 ▶); Soekamto *et al.* (2003 ▶). For the synthesis of the starting material 2-ethyl-3-(4-fluoro­phenyl­sulfan­yl)-5,7-dimethyl-1-benzo­furan, see: Choi *et al.* (1999 ▶). For a related structure, see: Choi *et al.* (2010 ▶).
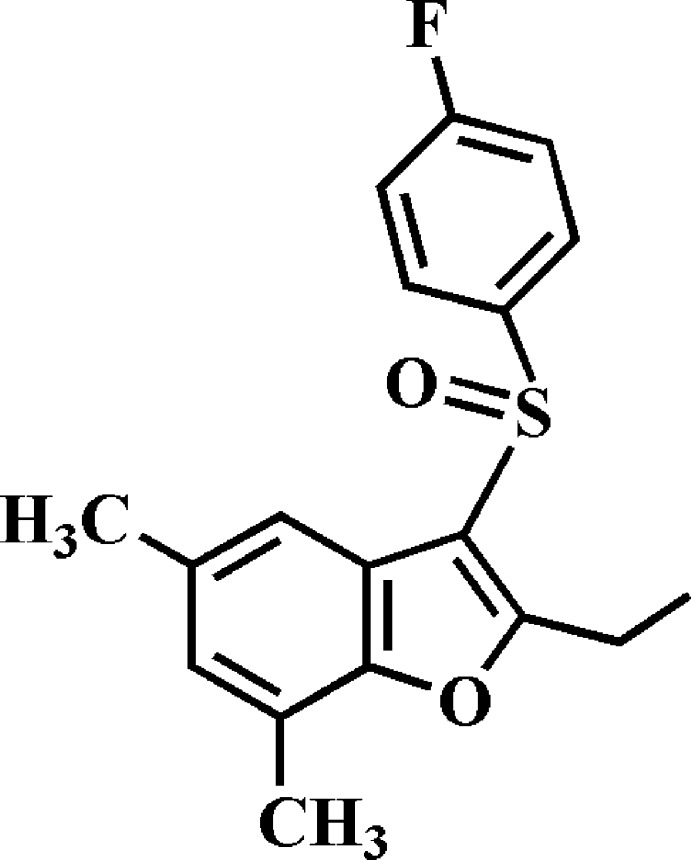


## Experimental   

### Crystal data  

C_18_H_17_FO_2_S
*M* *_r_* = 316.38
Triclinic, 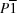

*a* = 9.1523 (2) Å
*b* = 9.5503 (2) Å
*c* = 10.3099 (2) Å
α = 65.666 (1)°
β = 81.636 (1)°
γ = 70.782 (1)°
*V* = 775.29 (3) Å^3^
*Z* = 2
Mo *K*α radiation
μ = 0.22 mm^−1^
*T* = 173 K
0.45 × 0.41 × 0.27 mm

### Data collection  

Bruker SMART APEXII CCD diffractometer
Absorption correction: multi-scan (*SADABS*; Bruker, 2009 ▶) *T*_min_ = 0.907, *T*_max_ = 0.942
14476 measured reflections
3874 independent reflections
3481 reflections with *I* > 2σ(*I*)
*R*_int_ = 0.028

### Refinement  

*R*[*F* ^2^ > 2σ(*F* ^2^)] = 0.063
*wR*(*F* ^2^) = 0.170
*S* = 1.08
3874 reflections
209 parameters
17 restraints
H-atom parameters constrained
Δρ_max_ = 0.94 e Å^−3^
Δρ_min_ = −1.64 e Å^−3^

### 

Data collection: *APEX2* (Bruker, 2009 ▶); cell refinement: *SAINT* (Bruker, 2009 ▶); data reduction: *SAINT*; program(s) used to solve structure: *SHELXS97* (Sheldrick, 2008 ▶); program(s) used to refine structure: *SHELXL97* (Sheldrick, 2008 ▶); molecular graphics: *ORTEP-3 for Windows* (Farrugia, 2012 ▶) and *DIAMOND* (Brandenburg, 1998 ▶); software used to prepare material for publication: *SHELXL97*.

## Supplementary Material

Crystal structure: contains datablock(s) I. DOI: 10.1107/S1600536814019023/hb7276sup1.cif

Structure factors: contains datablock(s) I. DOI: 10.1107/S1600536814019023/hb7276Isup2.hkl

Supporting information file. DOI: 10.1107/S1600536814019023/hb7276Isup3.cml

**Fig. 1.**
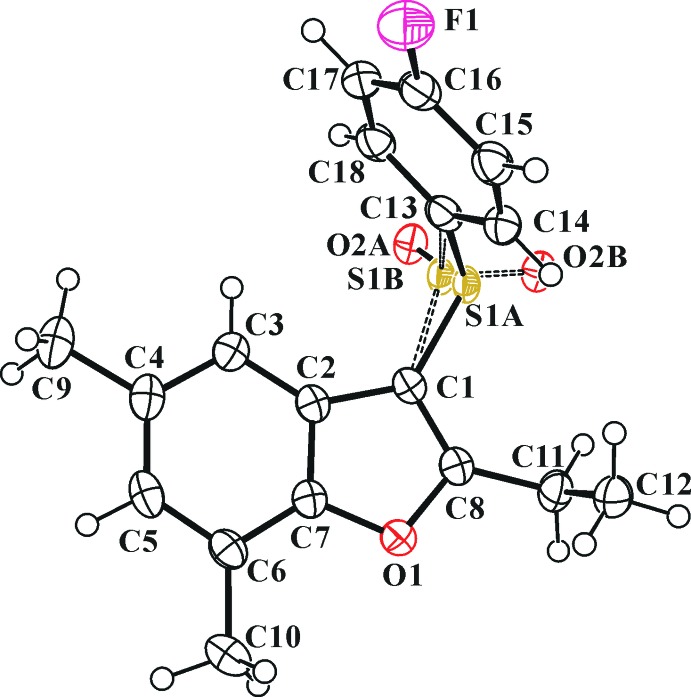
The molecular structure of the title compound with displacement ellipsoids drawn at the 50% probability level. The S1 and O2 atoms of the sulfinyl group are disordered over two positions with site-occupancy factors, from refinement of 0.797 (3) (part A) and 0.213 (3) (part B).

**Fig. 2.**
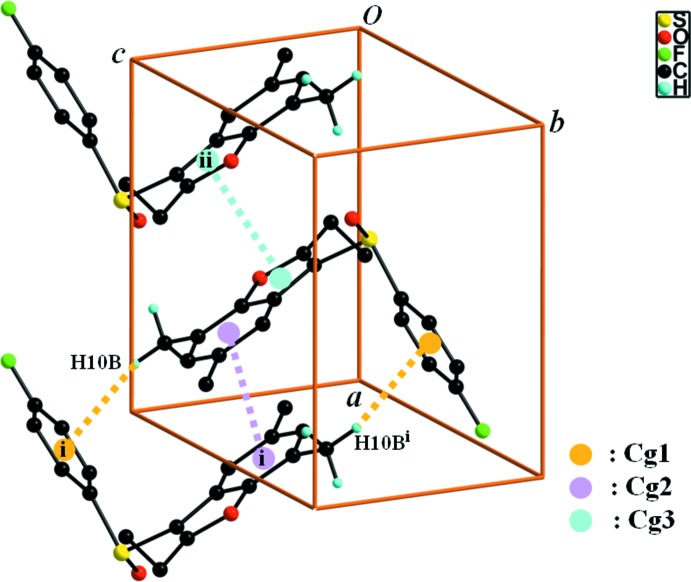
A view of the C–H···π and π–π interactions (dotted lines) in the crystal structure of the title compound. H atoms non-participating in hydrogen-bonding and disordered part B atoms were omitted for clarity. [Symmetry codes: (i) -x+2, -y, -z+1; (ii) -x+1, -y, -z+1.]

. DOI: 10.1107/S1600536814019023/hb7276fig1.tif
The mol­ecular structure of the title compound with displacement ellipsoids drawn at the 50% probability level. The S1 and O2 atoms of the sulfinyl group are disordered over two positions with site-occupancy factors, from refinement of 0.797 (3) (part A) and 0.213 (3) (part B).

x y z x y z . DOI: 10.1107/S1600536814019023/hb7276fig2.tif
A view of the C–H⋯π and π–π inter­actions (dotted lines) in the crystal structure of the title compound. H atoms non-participating in hydrogen-bonding and disordered part B atoms were omitted for clarity. [Symmetry codes: (i) −*x* + 2, −*y*, −*z* + 1; (ii) −*x* + 1, −*y*, −*z* + 1.]

CCDC reference: 1020650

Additional supporting information:  crystallographic information; 3D view; checkCIF report

## Figures and Tables

**Table 1:** Hydrogen-bond geometry (Å, °) *Cg*1 is the centroid of the C13–C18 phenyl ring.

*D*—H⋯*A*	*D*—H	H⋯*A*	*D*⋯*A*	*D*—H⋯*A*
C10—H10*B*⋯*Cg*1^i^	0.98	2.89	3.822 (2)	159

Symmetry code: (i) 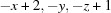.

## supplementary crystallographic information

### S1. Comment

Benzofuran compounds show various pharmacological properties such as antibacterial and antifungal, antitumor and antiviral, antimicrobial activities (Aslam *et al.*. 2009, Galal *et al.*, 2009, Khan *et al.*, 2005), and potential inhibitor of β-amyloid aggregation (Howlett *et al.*, 1999, Ono *et al.*, 2002). These benzofuran compounds are widely occurring in nature (Akgul & Anil, 2003, Soekamto *et al.*, 2003). As a part of our ongoing project of 3-(4-fluorophenylsulfinyl)-5,7-dimethyl-1-benzofuran derivatives containing methyl substituent in 2-position (Choi *et al.*, 2010), we report herein on the crystal structure of the title compound.

In the title molecule (Fig. 1), the benzofuran unit is essentially planar, with a mean deviation of 0.004 (2) Å from the least-squares plane defined by the nine constituent atoms. The 4-fluorophenyl ring is essentially planar, with a mean deviation of 0.005 (2) Å from the least-squares plane defined by the six constituent atoms. In the sulfinyl group, the S1 and O2 atoms are disordered over two positions with site-occupancy factors, from refinement of 0.797 (3) (part A) and 0.213 (3) (part B). The dihedral angle formed by the benzofuran ring and the 4-fluorophenyl ring is 86.38 (6)°. In the crystal structure (Fig. 2), molecules are linked via pairs of C–H···π interactions (Table 1, Cg1 is the C13-C18 4-fluorophenyl ring) into inversion-related dimers. These dimers are further linked by π–π interactions between the benzene rings of neighbouring molecules with a Cg2···Cg2_i_ distance of 3.715 (3) Å and an interplanar distance of 3.464 (3) Å resulting in a slippage of 1.342 (3) Å (Cg2 is the C2-C7 benzene ring), and between the furan rings of neighbouring molecules with a Cg3···Cg3^ii^ distance of 3.598 (3) Å and an interplanar distance of 3.507 (3) Å resulting in a slippage of 0.804 (3) Å (Cg3 is the C1/C2/C7/O1/C8 furan ring). The molecules are stacked along the *a*-axis direction.

### S2. Experimental

The starting material 2-ethyl-3-(4-fluorophenylsulfanyl)-5,7-dimethyl-1-benzofuran was prepared by literature method (Choi *et al.* 1999). 3-Chloroperoxybenzoic acid (77%, 224 mg, 1.0 mmol) was added in small portions to a stirred solution of 2-ethyl-3-(4-fluorophenylsulfanyl)-5,7-dimethyl-1-benzofuran (270 mg, 0.9 mmol) in dichloromethane (30 ml) at 273 K. After being stirred at room temperature for 8h, the mixture was washed with saturated sodium bicarbonate solution (2 X × 10 ml) and the organic layer was separated, dried over magnesium sulfate, filtered and concentrated at reduced pressure. The residue was purified by column chromatography (hexane-ethyl acetate, 2:1 *v*/*v*) to afford the title compound as a colorless solid [yield 74% (234 mg); m.p. 378-379 K; *R*_f_ = 0.59 (hexane-ethyl acetate, 2:1 *v*/*v*)]. Colourless blocks were prepared by slow evaporation of a solution of the title compound (20 mg) in acetone (15 ml) at room temperature.

### S3. Refinement

All H atoms were positioned geometrically and refined using a riding model, with C–H = 0.95 Å for aryl and 0.98 Å for methylene and 0.99 Å for methyl H atoms, respectively. *U*_iso_ (H) = 1.2*U*_eq_ (C) for aryl and methylene, and 1.5*U*_eq_ (C) for methyl H atoms. The positions of methyl and methylene hydrogens were optimized using the SHELXL-97's command AFIX 137 (Sheldrick, 2008).The S1 and O2 atoms of the sulfinyl group is disordered over two positions with site-occupancy factors, from refinement of 0.797 (3) (part A) and 0.213 (3) (part B). The distance of equivalent S═O, C(pheny)–S and C(furan)–S pairs were restrained to 1.488 (1), 1.762 (1) and 1.788 (1) Å using command DFIX and DELU, and displacement ellipsoids of S1 and O2 set were restrained using SHELXL command EADP, respectively.

### Crystal data

**Table:**

C_18_H_17_FO_2_S	*V* = 775.29 (3) Å^3^
*M**_r_* = 316.38	*Z* = 2
Triclinic, *P*1	*F*(000) = 332
Hall symbol: -P 1	*D*_x_ = 1.355 Mg m^−^^3^
*a* = 9.1523 (2) Å	Melting point = 379–378 K
*b* = 9.5503 (2) Å	Mo *K*α radiation, λ = 0.71073 Å
*c* = 10.3099 (2) Å	µ = 0.22 mm^−^^1^
α = 65.666 (1)°	*T* = 173 K
β = 81.636 (1)°	Block, colourless
γ = 70.782 (1)°	0.45 × 0.41 × 0.27 mm

### Data collection

**Table:**

Bruker SMART APEXII CCD diffractometer	3874 independent reflections
Radiation source: rotating anode	3481 reflections with *I* > 2σ(*I*)
Graphite multilayer monochromator	*R*_int_ = 0.028
Detector resolution: 10.0 pixels mm^-1^	θ_max_ = 28.5°, θ_min_ = 2.2°
φ and ω scans	*h* = −12→12
Absorption correction: multi-scan (*SADABS*; Bruker, 2009)	*k* = −12→12
*T*_min_ = 0.907, *T*_max_ = 0.942	*l* = −13→13
14476 measured reflections	

### Refinement

**Table:**

Refinement on *F*^2^	Primary atom site location: structure-invariant direct methods
Least-squares matrix: full	Secondary atom site location: difference Fourier map
*R*[*F*^2^ > 2σ(*F*^2^)] = 0.063	Hydrogen site location: difference Fourier map
*wR*(*F*^2^) = 0.170	H-atom parameters constrained
*S* = 1.08	*w* = 1/[σ^2^(*F*_o_^2^) + (0.0799*P*)^2^ + 0.8614*P*] where *P* = (*F*_o_^2^ + 2*F*_c_^2^)/3
3874 reflections	(Δ/σ)_max_ < 0.001
209 parameters	Δρ_max_ = 0.94 e Å^−^^3^
17 restraints	Δρ_min_ = −1.64 e Å^−^^3^

### Special details

**Table:**

Experimental. ^1^H NMR (δ p.p.m., CDCl_3_, 400 Hz): 7.62-7.67 (m, 2H), 7.15-7.21 (m, 2H), 6.85 (s, 2H), 3.13 (q, J =7.52 Hz, 2H), 2.43 (s, 3H), 2.24 (s, 3H), 1.44 (t, J = 7.52 Hz, 3H).
Geometry. All esds (except the esd in the dihedral angle between two l.s. planes) are estimated using the full covariance matrix. The cell esds are taken into account individually in the estimation of esds in distances, angles and torsion angles; correlations between esds in cell parameters are only used when they are defined by crystal symmetry. An approximate (isotropic) treatment of cell esds is used for estimating esds involving l.s. planes.
Refinement. Refinement of F^2^ against ALL reflections. The weighted R-factor wR and goodness of fit S are based on F^2^, conventional R-factors R are based on F, with F set to zero for negative F^2^. The threshold expression of F^2^ > 2sigma(F^2^) is used only for calculating R-factors(gt) etc. and is not relevant to the choice of reflections for refinement. R-factors based on F^2^ are statistically about twice as large as those based on F, and R- factors based on ALL data will be even larger.

### Fractional atomic coordinates and isotropic or equivalent isotropic displacement parameters (Å^2^)

**Table:**

	*x*	*y*	*z*	*U*_iso_*/*U*_eq_	Occ. (<1)
S1A	0.46524 (7)	0.39880 (7)	0.27750 (7)	0.0245 (2)	0.797 (3)
O2A	0.3828 (2)	0.4499 (2)	0.39425 (17)	0.0291 (4)	0.797 (3)
S1B	0.47017 (19)	0.40209 (16)	0.3147 (3)	0.0245 (2)	0.20
O2B	0.3618 (7)	0.4565 (9)	0.1973 (6)	0.0291 (4)	0.20
F1	0.9355 (2)	0.7389 (2)	0.05373 (19)	0.0497 (4)	
O1	0.69201 (18)	−0.05783 (19)	0.39017 (17)	0.0278 (4)	
C1	0.5858 (2)	0.20046 (14)	0.3595 (2)	0.0255 (4)	
C2	0.6924 (2)	0.1271 (3)	0.4764 (2)	0.0250 (4)	
C3	0.7407 (3)	0.1777 (3)	0.5674 (2)	0.0278 (5)	
H3	0.6994	0.2857	0.5605	0.033*	
C4	0.8512 (3)	0.0653 (3)	0.6687 (2)	0.0312 (5)	
C5	0.9101 (3)	−0.0934 (3)	0.6775 (2)	0.0324 (5)	
H5	0.9856	−0.1675	0.7476	0.039*	
C6	0.8642 (3)	−0.1485 (3)	0.5895 (2)	0.0286 (5)	
C7	0.7543 (2)	−0.0323 (3)	0.4904 (2)	0.0262 (4)	
C8	0.5892 (2)	0.0857 (3)	0.3131 (2)	0.0264 (4)	
C9	0.9111 (3)	0.1158 (4)	0.7667 (3)	0.0416 (6)	
H9A	0.8504	0.2264	0.7538	0.062*	
H9B	1.0201	0.1105	0.7439	0.062*	
H9C	0.9016	0.0434	0.8657	0.062*	
C10	0.9286 (3)	−0.3185 (3)	0.5983 (3)	0.0381 (6)	
H10A	0.8436	−0.3642	0.6110	0.057*	
H10B	0.9986	−0.3831	0.6794	0.057*	
H10C	0.9857	−0.3192	0.5104	0.057*	
C11	0.5104 (3)	0.0881 (3)	0.1959 (3)	0.0334 (5)	
H11A	0.4131	0.1784	0.1735	0.040*	
H11B	0.4830	−0.0132	0.2281	0.040*	
C12	0.6102 (3)	0.1066 (3)	0.0612 (3)	0.0388 (6)	
H12A	0.6319	0.2102	0.0252	0.058*	
H12B	0.5551	0.1027	−0.0113	0.058*	
H12C	0.7078	0.0189	0.0832	0.058*	
C13	0.61273 (19)	0.4976 (2)	0.2173 (2)	0.0284 (4)	
C14	0.6924 (3)	0.4950 (3)	0.0925 (2)	0.0314 (5)	
H14	0.6723	0.4362	0.0455	0.038*	
C15	0.8004 (3)	0.5770 (3)	0.0365 (3)	0.0334 (5)	
H15	0.8551	0.5759	−0.0489	0.040*	
C16	0.8272 (3)	0.6608 (3)	0.1073 (3)	0.0333 (5)	
C17	0.7502 (3)	0.6671 (3)	0.2305 (3)	0.0352 (5)	
H17	0.7718	0.7258	0.2769	0.042*	
C18	0.6402 (3)	0.5856 (3)	0.2857 (3)	0.0329 (5)	
H18	0.5839	0.5898	0.3698	0.040*	

### Atomic displacement parameters (Å^2^)

**Table:**

	*U*^11^	*U*^22^	*U*^33^	*U*^12^	*U*^13^	*U*^23^
S1A	0.0258 (3)	0.0285 (3)	0.0181 (4)	−0.0026 (2)	−0.0041 (2)	−0.0109 (2)
O2A	0.0216 (8)	0.0372 (10)	0.0300 (9)	−0.0030 (7)	−0.0004 (5)	−0.0190 (8)
S1B	0.0258 (3)	0.0285 (3)	0.0181 (4)	−0.0026 (2)	−0.0041 (2)	−0.0109 (2)
O2B	0.0216 (8)	0.0372 (10)	0.0300 (9)	−0.0030 (7)	−0.0004 (5)	−0.0190 (8)
F1	0.0540 (10)	0.0477 (9)	0.0512 (10)	−0.0292 (8)	0.0032 (8)	−0.0135 (8)
O1	0.0282 (8)	0.0260 (7)	0.0279 (8)	−0.0071 (6)	−0.0024 (6)	−0.0094 (6)
C1	0.0242 (10)	0.0257 (7)	0.0229 (9)	−0.0056 (6)	−0.0007 (7)	−0.0073 (8)
C2	0.0224 (9)	0.0283 (10)	0.0218 (9)	−0.0083 (8)	0.0016 (7)	−0.0073 (8)
C3	0.0278 (10)	0.0322 (11)	0.0253 (10)	−0.0119 (9)	0.0034 (8)	−0.0118 (9)
C4	0.0280 (11)	0.0438 (13)	0.0237 (10)	−0.0168 (10)	0.0018 (8)	−0.0110 (9)
C5	0.0266 (11)	0.0389 (12)	0.0236 (10)	−0.0112 (9)	−0.0033 (8)	−0.0025 (9)
C6	0.0257 (10)	0.0282 (10)	0.0257 (10)	−0.0087 (8)	0.0010 (8)	−0.0045 (8)
C7	0.0245 (10)	0.0291 (10)	0.0238 (10)	−0.0097 (8)	0.0006 (8)	−0.0083 (8)
C8	0.0240 (10)	0.0291 (10)	0.0240 (10)	−0.0081 (8)	0.0000 (8)	−0.0083 (8)
C9	0.0429 (14)	0.0578 (17)	0.0311 (12)	−0.0218 (13)	−0.0025 (10)	−0.0183 (12)
C10	0.0338 (12)	0.0280 (11)	0.0403 (13)	−0.0046 (9)	−0.0036 (10)	−0.0043 (10)
C11	0.0333 (12)	0.0385 (12)	0.0318 (12)	−0.0107 (10)	−0.0057 (9)	−0.0152 (10)
C12	0.0540 (16)	0.0366 (13)	0.0276 (11)	−0.0157 (11)	−0.0010 (11)	−0.0126 (10)
C13	0.0288 (10)	0.0226 (10)	0.0265 (10)	−0.0019 (7)	−0.0031 (8)	−0.0060 (8)
C14	0.0376 (12)	0.0295 (11)	0.0264 (10)	−0.0074 (9)	−0.0028 (9)	−0.0113 (9)
C15	0.0375 (12)	0.0328 (11)	0.0259 (11)	−0.0084 (10)	0.0011 (9)	−0.0097 (9)
C16	0.0363 (12)	0.0262 (11)	0.0322 (12)	−0.0087 (9)	−0.0043 (9)	−0.0057 (9)
C17	0.0461 (14)	0.0261 (11)	0.0330 (12)	−0.0053 (10)	−0.0081 (10)	−0.0129 (9)
C18	0.0392 (12)	0.0263 (10)	0.0267 (11)	−0.0006 (9)	−0.0011 (9)	−0.0108 (9)

### Geometric parameters (Å, º)

**Table:**

S1A—O2B	1.180 (3)	C8—C11	1.484 (3)
S1A—O2A	1.4943 (9)	C9—H9A	0.9800
S1A—C1	1.7597 (10)	C9—H9B	0.9800
S1A—C13	1.7918 (10)	C9—H9C	0.9800
O2A—S1B	1.182 (2)	C10—H10A	0.9800
S1B—O2B	1.4869 (10)	C10—H10B	0.9800
S1B—C1	1.7633 (10)	C10—H10C	0.9800
S1B—C13	1.7865 (10)	C11—C12	1.527 (4)
F1—C16	1.353 (3)	C11—H11A	0.9900
O1—C8	1.366 (3)	C11—H11B	0.9900
O1—C7	1.387 (3)	C12—H12A	0.9800
C1—C8	1.355 (3)	C12—H12B	0.9800
C1—C2	1.445 (3)	C12—H12C	0.9800
C2—C7	1.391 (3)	C13—C14	1.389 (3)
C2—C3	1.395 (3)	C13—C18	1.395 (3)
C3—C4	1.391 (3)	C14—C15	1.376 (4)
C3—H3	0.9500	C14—H14	0.9500
C4—C5	1.399 (4)	C15—C16	1.377 (4)
C4—C9	1.513 (3)	C15—H15	0.9500
C5—C6	1.388 (3)	C16—C17	1.374 (4)
C5—H5	0.9500	C17—C18	1.388 (4)
C6—C7	1.384 (3)	C17—H17	0.9500
C6—C10	1.502 (3)	C18—H18	0.9500
			
O2B—S1A—O2A	98.8 (4)	H9A—C9—H9B	109.5
O2B—S1A—C1	132.7 (4)	C4—C9—H9C	109.5
O2A—S1A—C1	106.79 (11)	H9A—C9—H9C	109.5
O2B—S1A—C13	113.2 (4)	H9B—C9—H9C	109.5
O2A—S1A—C13	104.60 (11)	C6—C10—H10A	109.5
C1—S1A—C13	98.06 (10)	C6—C10—H10B	109.5
S1B—O2A—S1A	11.31 (14)	H10A—C10—H10B	109.5
O2A—S1B—O2B	99.1 (3)	C6—C10—H10C	109.5
O2A—S1B—C1	124.2 (2)	H10A—C10—H10C	109.5
O2B—S1B—C1	112.3 (3)	H10B—C10—H10C	109.5
O2A—S1B—C13	121.5 (2)	C8—C11—C12	112.6 (2)
O2B—S1B—C13	99.4 (3)	C8—C11—H11A	109.1
C1—S1B—C13	98.13 (12)	C12—C11—H11A	109.1
S1A—O2B—S1B	11.63 (11)	C8—C11—H11B	109.1
C8—O1—C7	106.69 (17)	C12—C11—H11B	109.1
C8—C1—C2	108.00 (14)	H11A—C11—H11B	107.8
C8—C1—S1A	120.39 (16)	C11—C12—H12A	109.5
C2—C1—S1A	131.60 (16)	C11—C12—H12B	109.5
C8—C1—S1B	132.68 (19)	H12A—C12—H12B	109.5
C2—C1—S1B	119.05 (19)	C11—C12—H12C	109.5
S1A—C1—S1B	13.29 (10)	H12A—C12—H12C	109.5
C7—C2—C3	119.4 (2)	H12B—C12—H12C	109.5
C7—C2—C1	104.31 (18)	C14—C13—C18	120.08 (16)
C3—C2—C1	136.2 (2)	C14—C13—S1B	130.30 (19)
C4—C3—C2	118.0 (2)	C18—C13—S1B	109.61 (19)
C4—C3—H3	121.0	C14—C13—S1A	117.84 (16)
C2—C3—H3	121.0	C18—C13—S1A	121.90 (17)
C3—C4—C5	120.1 (2)	S1B—C13—S1A	13.08 (10)
C3—C4—C9	119.9 (2)	C15—C14—C13	120.4 (2)
C5—C4—C9	120.0 (2)	C15—C14—H14	119.8
C6—C5—C4	123.6 (2)	C13—C14—H14	119.8
C6—C5—H5	118.2	C14—C15—C16	118.4 (2)
C4—C5—H5	118.2	C14—C15—H15	120.8
C7—C6—C5	114.2 (2)	C16—C15—H15	120.8
C7—C6—C10	122.3 (2)	F1—C16—C17	118.7 (2)
C5—C6—C10	123.5 (2)	F1—C16—C15	118.4 (2)
C6—C7—O1	124.8 (2)	C17—C16—C15	122.9 (2)
C6—C7—C2	124.7 (2)	C16—C17—C18	118.5 (2)
O1—C7—C2	110.49 (18)	C16—C17—H17	120.8
C1—C8—O1	110.49 (18)	C18—C17—H17	120.8
C1—C8—C11	133.2 (2)	C17—C18—C13	119.7 (2)
O1—C8—C11	116.23 (19)	C17—C18—H18	120.2
C4—C9—H9A	109.5	C13—C18—H18	120.2
C4—C9—H9B	109.5		
			
O2B—S1A—O2A—S1B	−169.5 (5)	C10—C6—C7—C2	−179.1 (2)
C1—S1A—O2A—S1B	50.7 (4)	C8—O1—C7—C6	−179.7 (2)
C13—S1A—O2A—S1B	−52.6 (4)	C8—O1—C7—C2	−0.5 (2)
S1A—O2A—S1B—O2B	8.3 (4)	C3—C2—C7—C6	−0.5 (3)
S1A—O2A—S1B—C1	−116.6 (4)	C1—C2—C7—C6	179.3 (2)
S1A—O2A—S1B—C13	115.3 (4)	C3—C2—C7—O1	−179.77 (18)
O2A—S1A—O2B—S1B	8.1 (4)	C1—C2—C7—O1	0.1 (2)
C1—S1A—O2B—S1B	130.9 (7)	C2—C1—C8—O1	−0.7 (2)
C13—S1A—O2B—S1B	−102.0 (5)	S1A—C1—C8—O1	179.05 (14)
O2A—S1B—O2B—S1A	−169.7 (5)	S1B—C1—C8—O1	−174.62 (18)
C1—S1B—O2B—S1A	−36.8 (6)	C2—C1—C8—C11	−178.0 (2)
C13—S1B—O2B—S1A	66.1 (5)	S1A—C1—C8—C11	1.8 (4)
O2B—S1A—C1—C8	13.3 (6)	S1B—C1—C8—C11	8.1 (4)
O2A—S1A—C1—C8	133.10 (19)	C7—O1—C8—C1	0.8 (2)
C13—S1A—C1—C8	−118.93 (19)	C7—O1—C8—C11	178.54 (18)
O2B—S1A—C1—C2	−166.9 (5)	C1—C8—C11—C12	96.1 (3)
O2A—S1A—C1—C2	−47.2 (2)	O1—C8—C11—C12	−81.0 (3)
C13—S1A—C1—C2	60.8 (2)	O2A—S1B—C13—C14	−158.4 (3)
O2B—S1A—C1—S1B	−146.0 (6)	O2B—S1B—C13—C14	−51.6 (4)
O2A—S1A—C1—S1B	−26.3 (2)	C1—S1B—C13—C14	62.8 (3)
C13—S1A—C1—S1B	81.7 (2)	O2A—S1B—C13—C18	21.6 (3)
O2A—S1B—C1—C8	115.2 (3)	O2B—S1B—C13—C18	128.4 (3)
O2B—S1B—C1—C8	−3.8 (4)	C1—S1B—C13—C18	−117.2 (2)
C13—S1B—C1—C8	−107.5 (3)	O2A—S1B—C13—S1A	−139.2 (4)
O2A—S1B—C1—C2	−58.2 (3)	O2B—S1B—C13—S1A	−32.4 (4)
O2B—S1B—C1—C2	−177.2 (4)	C1—S1B—C13—S1A	81.9 (2)
C13—S1B—C1—C2	79.1 (2)	O2B—S1A—C13—C14	−62.9 (4)
O2A—S1B—C1—S1A	139.6 (4)	O2A—S1A—C13—C14	−169.35 (18)
O2B—S1B—C1—S1A	20.6 (4)	C1—S1A—C13—C14	80.87 (19)
C13—S1B—C1—S1A	−83.1 (2)	O2B—S1A—C13—C18	112.2 (4)
C8—C1—C2—C7	0.4 (2)	O2A—S1A—C13—C18	5.7 (2)
S1A—C1—C2—C7	−179.35 (18)	C1—S1A—C13—C18	−104.0 (2)
S1B—C1—C2—C7	175.26 (16)	O2B—S1A—C13—S1B	133.5 (4)
C8—C1—C2—C3	−179.8 (2)	O2A—S1A—C13—S1B	27.1 (2)
S1A—C1—C2—C3	0.4 (4)	C1—S1A—C13—S1B	−82.7 (2)
S1B—C1—C2—C3	−4.9 (4)	C18—C13—C14—C15	0.9 (3)
C7—C2—C3—C4	0.6 (3)	S1B—C13—C14—C15	−179.1 (2)
C1—C2—C3—C4	−179.2 (2)	S1A—C13—C14—C15	176.07 (18)
C2—C3—C4—C5	−0.2 (3)	C13—C14—C15—C16	0.2 (4)
C2—C3—C4—C9	178.0 (2)	C14—C15—C16—F1	178.7 (2)
C3—C4—C5—C6	−0.1 (4)	C14—C15—C16—C17	−0.5 (4)
C9—C4—C5—C6	−178.4 (2)	F1—C16—C17—C18	−179.4 (2)
C4—C5—C6—C7	0.2 (3)	C15—C16—C17—C18	−0.2 (4)
C4—C5—C6—C10	179.5 (2)	C16—C17—C18—C13	1.2 (3)
C5—C6—C7—O1	179.3 (2)	C14—C13—C18—C17	−1.6 (3)
C10—C6—C7—O1	0.0 (3)	S1B—C13—C18—C17	178.40 (19)
C5—C6—C7—C2	0.1 (3)	S1A—C13—C18—C17	−176.58 (17)

### Hydrogen-bond geometry (Å, º)

Cg1 is the centroid of the C13–C18 phenyl ring.

**Table:**

*D*—H···*A*	*D*—H	H···*A*	*D*···*A*	*D*—H···*A*
C10—H10*B*···*Cg*1^i^	0.98	2.89	3.822 (2)	159

Symmetry code: (i) −*x*+2, −*y*, −*z*+1.
