# Supplementary figures and images for: Crystal structure of 2-ethyl-3-(4-fluoro­phenyl­sulfin­yl)-5,7-dimethyl-1-benzo­furan
Source: Acta Crystallogr Sect E Struct Rep Online. 2014 Aug 30;70(Pt 9):o1058–9. doi: 10.1107/S1600536814019023 (PMC4186098; doi:10.1107/S1600536814019023)

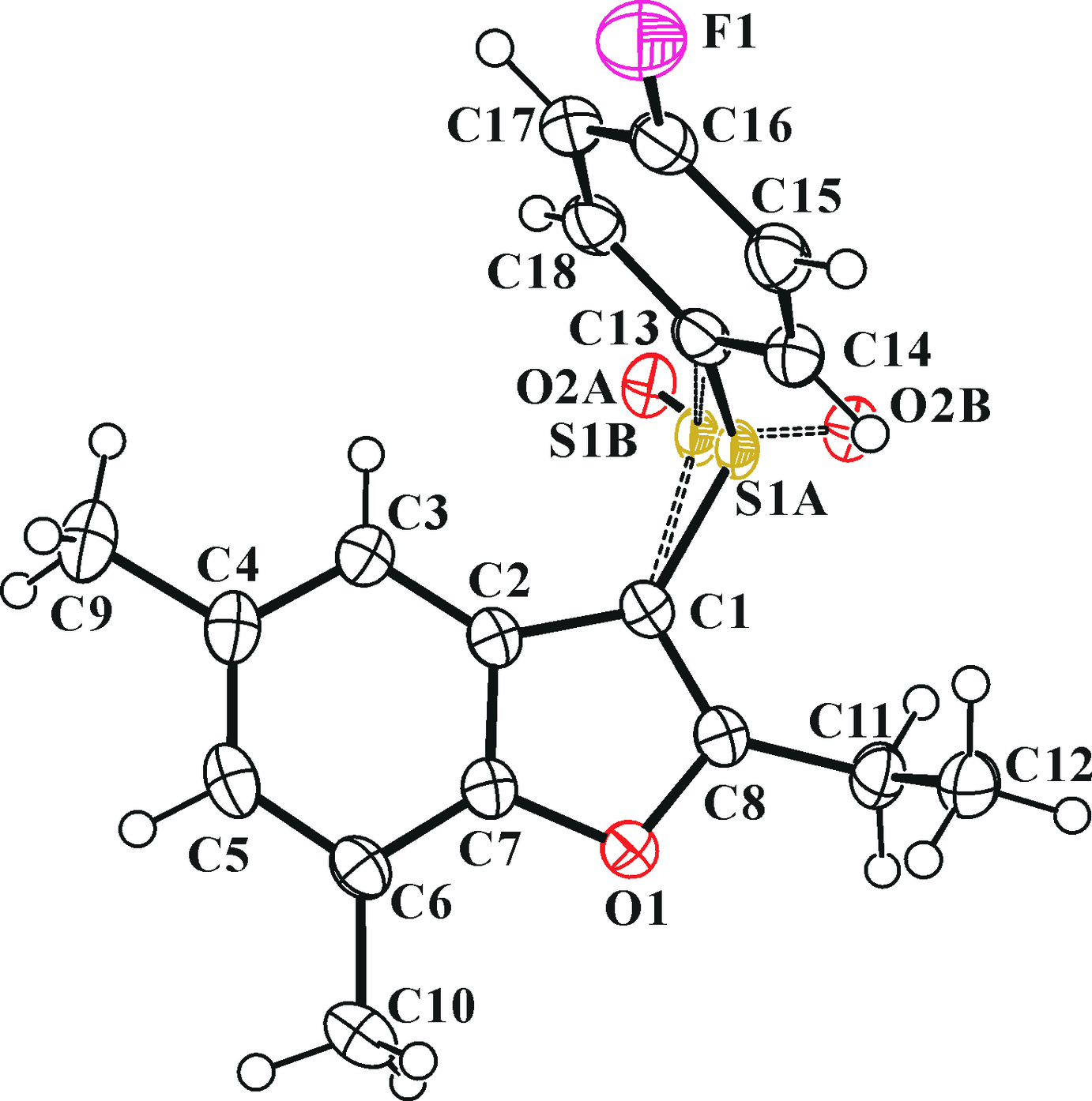

Supplement: Supplementary file 4 [file e-70-o1058-fig1.tif]

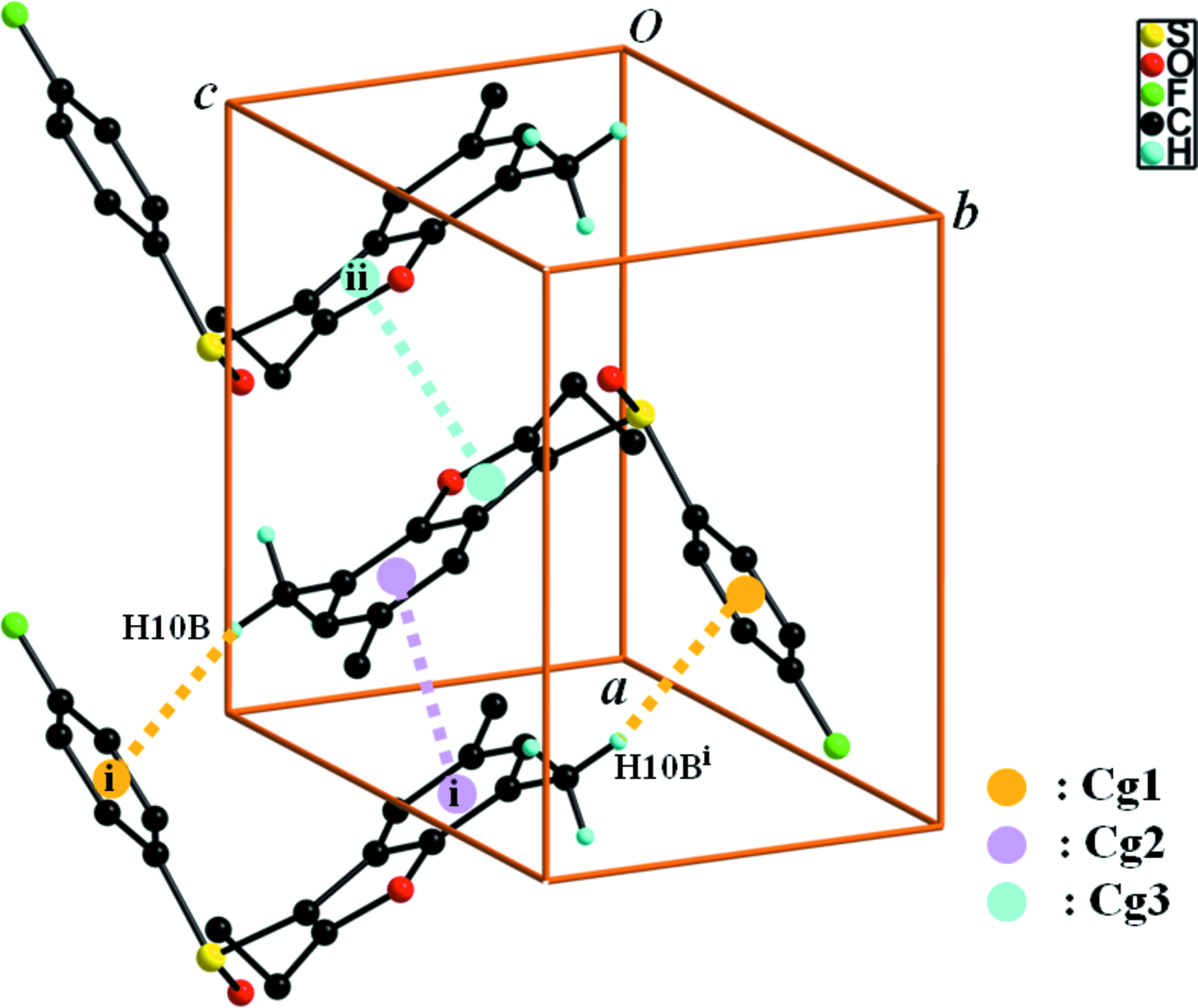

Supplement: Supplementary file 5 [file e-70-o1058-fig2.tif]
